# Insights into the relationship between anxiety and attitudes toward artificial intelligence among nursing students

**DOI:** 10.1186/s12912-025-03490-2

**Published:** 2025-07-01

**Authors:** Ahmad Ayed, Moath Abu Ejheisheh, Rasmieh Al-Amer, Ibrahim Aqtam, Amira Mohammed Ali, Elham H. Othman, Mosaab Farajallah, Jamal Qaddumi, Ahmad Batran

**Affiliations:** 1 Faculty of Nursing, Arab American University, Jenin, Palestine; 2 Department of Nursing, Faculty of Allied Medical Sciences, Palestine Ahliya University, Bethlehem, Palestine; 3 Faculty of Nursing, Yarmouk University, Irbid, Jordan; 4 Department of Nursing, Ibn Sina College for Health Professions, Nablus University for Vocational and Technical Education, Nablus, Palestine; 5 Department of Psychiatric Nursing and Mental Health, Faculty of Nursing, Alexandria University, Smouha, Alexandria 21527 Egypt; 6 Faculty of Nursing, Applied Science Private University, Amman, Jordan; 7 Faculty of Medicine and Health Sciences, An-Najah National University, Nablus, Palestine

**Keywords:** Artificial intelligence, Attitude, Anxiety, Nursing students, Palestine

## Abstract

**Background:**

Artificial Intelligence (AI) integration in healthcare education represents a critical technological advancement that requires careful examination of student preparedness and acceptance. In the Palestinian context, limited research exists on nursing students’ psychological responses to AI implementation, despite growing global emphasis on AI competency in healthcare professions. Understanding the relationship between anxiety and attitudes toward AI is essential for developing effective educational strategies that can facilitate successful technology adoption while addressing cultural and contextual barriers specific to the Palestinian healthcare education environment.

**Introduction:**

Artificial Intelligence (AI) integration in nursing education remains underexplored in the Palestinian context, where limited research addresses students’ anxiety and attitudes toward AI. This study examines this relationship to fill a critical gap and inform culturally relevant strategies for AI adoption in healthcare education.

**Methods:**

A cross-sectional study was conducted among 264 nursing students at Palestine Ahliya University (2024–2025). Validated scales (AI Anxiety Scale, SATAI) assessed anxiety and attitudes. We analyzed data via correlation and regression using SPSS v26.

**Results:**

High AI anxiety (mean = 80.3, SD = 9.4) contrasted with positive attitudes (mean = 114.3, SD = 12.8). Regression identified attitude as the strongest predictor of anxiety (B = 5.171, *p* < .001), alongside younger age, female gender, and non-use of AI. Academic year and AI education showed no significant effects.

**Conclusion:**

Negative attitudes and limited AI exposure drive anxiety, particularly among younger females and non-users. To mitigate this, we recommend integrating AI literacy modules into curricula, fostering hands-on AI experiences, and designing gender-sensitive training. These findings emphasize the urgency of addressing sociocultural and educational barriers to AI readiness in Palestinian nursing education.

**Clinical trial number:**

Not applicable.

**Supplementary Information:**

The online version contains supplementary material available at 10.1186/s12912-025-03490-2.

## Introduction

The rapid advancement of artificial intelligence (AI) in healthcare has created an imperative for nursing education to adapt and prepare future professionals for AI-integrated clinical environments [1, 2]. Globally, AI technologies are transforming healthcare delivery through enhanced diagnostic capabilities, predictive analytics, and streamlined administrative processes [3, 4]. However, the successful integration of AI in healthcare settings depends critically on healthcare professionals’ readiness, competency, and psychological acceptance of these technologies [5, 6].

## Literature review and theoretical framework

Research in AI acceptance among healthcare students reveals complex relationships between technological exposure, anxiety levels, and behavioral intentions [7, 8]. The Technology Acceptance Model (TAM) suggests that perceived usefulness and ease of use are primary determinants of technology adoption [9], yet recent studies indicate that cultural, gender, and experiential factors significantly moderate these relationships [10, 11]. Studies from developed countries show that nursing students generally exhibit positive attitudes toward AI but experience varying levels of anxiety related to job displacement concerns and technological competency fears [12, 13].

In Middle Eastern contexts, research has identified unique challenges including limited technological infrastructure, cultural hesitancy toward autonomous systems, and gendered access to technology resources [14, 15]. Recent studies by Tarsuslu, Agaoglu, and Bas found that digital leadership significantly influences AI anxiety and attitudes among nurses, suggesting that institutional support and training quality are crucial moderating factors [16]. Similarly, regional investigations have highlighted the importance of culturally sensitive approaches to AI education that address local concerns while building technological confidence [17].

In healthcare, AI enhances clinical decision-making, improves diagnostic accuracy, and streamlines administrative processes [1, 2]. Despite global advancements, Palestinian nursing curricula rarely incorporate AI, and students’ readiness for AI-driven healthcare environments remains unexamined [18]. This gap is critical, as future healthcare professionals must develop AI competencies to ensure effective clinical integration [8]. Studies in neighboring Arab countries highlight cultural hesitancy and resource limitations as barriers to AI adoption [11], yet Palestinian-specific insights are absent.

## Problem definition and research gap

The rapid growth of AI in healthcare necessitates urgent attention to nursing students’ perceptions, particularly in regions lagging in technological infrastructure [6, 15]. While younger, technologically adept students globally show receptiveness to AI [19], older individuals or those with limited exposure exhibit hesitation. In Palestine, socioeconomic constraints, limited institutional support, and gendered access to technology further complicate AI readiness, a dimension overlooked in existing literature [14].

## Research questions and study contribution

This study addresses three research questions:

1. What is the relationship between AI anxiety and attitudes among Palestinian nursing students?
2. How do demographic factors (age, gender, AI use) predict AI-related anxiety?
3. What actionable strategies can policymakers and educators implement to reduce anxiety and foster AI adoption in nursing curricula?

By focusing on Palestine, this study fills a critical gap in the literature by providing culturally-specific insights into AI acceptance patterns in a resource-constrained, developing healthcare education context [5, 10]. The study’s contribution lies in its examination of the intersection between technological anxiety, cultural factors, and educational preparedness in a region where such research is virtually absent [15].

For instance, AI-assisted tools like ChatGPT are increasingly used in global nursing education [18], yet Palestinian institutions lack structured AI policies. Addressing this disparity requires empirical evidence to guide curriculum redesign and resource allocation. The findings aim to inform national strategies for AI integration, aligning with the Palestinian Ministry of Health’s 2030 Digital Transformation Agenda [20].

## Methods

### Study design and setting

A cross-sectional descriptive design was utilized to examine the relationship between anxiety and attitudes toward AI among nursing students in the understudied Palestinian context [7, 13]. The study was conducted at Palestine Ahliya University, a leading institution in nursing education in the West Bank. Data collection spanned September–November 2024 during the first academic semester.

### Population and sampling

The target population included nursing students enrolled in the BSN program (*N* = 800). Using Raosoft software, the sample size was calculated as 260 (95% CI, 5% margin of error, 50% response rate) [21]. To account for potential non-response, 285 questionnaires were distributed, yielding 264 completed responses (92.6% response rate). Convenience sampling was employed due to logistical constraints [22], but the high response rate and inclusion of students across all academic years (first to fourth) enhanced representativeness.

### Instruments

**1. AI Anxiety Scale**: The English version of the scale by Wang and Wang was administered to participants [23]. It comprises 21 items scored on a 5-point Likert scale (1 = Strongly Disagree to 5 = Strongly Agree), yielding a total score range of 21–105, where higher scores indicate greater anxiety. The scale was originally developed and validated in English-speaking contexts, and its cultural appropriateness for the Palestinian context was ensured through pilot testing with 30 nursing students [24, 25]. The pilot phase confirmed linguistic clarity and cultural relevance of all items. Additionally, the scale’s theoretical constructs (learning anxiety, job replacement concerns, sociotechnical blindness, and AI configuration anxiety) were found to be conceptually applicable to the Palestinian healthcare education environment. Reliability was confirmed through excellent internal consistency in this study (Cronbach’s α = 0.88). Scoring clarification resolved earlier discrepancies: the reported mean of 80.3 (SD = 9.4) aligns with the theoretical range (21–105), ensuring accuracy in interpreting anxiety levels. (See Supplementary Material A)

**2. Student Attitudes Toward AI (SATAI)**: The English version of the SATAI scale by Suh and Ahn was used [24]. It includes 26 items assessing cognitive, affective, and behavioral components of AI attitudes. Similar to the anxiety scale, the SATAI was pilot-tested for cultural appropriateness and linguistic clarity in the Palestinian context [23]. The three-dimensional structure (cognitive, affective, behavioral) was confirmed through exploratory factor analysis during the pilot phase, demonstrating adequate cultural validity for use in this population. Reliability: The scale showed strong reliability (Cronbach’s α = 0.92) in this study [5]. (See Supplementary Material A)

### Data collection and ethical compliance

Data were collected in nursing lab rooms by trained staff to minimize bias [26]. Questionnaires were administered in English, as all participants were proficient in the language due to prior academic training [27]. Ethical approval was obtained from Palestine Ahliya University (CAMS/BSN/48/2024) [1].

### Statistical analysis

Normality Testing was performed using the Shapiro-Wilk test, confirming that all variables adhered to a normal distribution (*p* >.05). For Regression Assumptions, multicollinearity was assessed via Variance Inflation Factor (VIF < 5) [11], while residual plots verified homoscedasticity and normality, and Durbin-Watson tests (DW = 1.92) indicated independence of residuals [15]. Analyses included Pearson’s correlation and multiple linear regression (SPSS v26) to identify significant predictors of AI anxiety [6].

## Results

### Demographic characteristics

Participants’ mean age was 21.1 years (SD = 1.3), with males (60.6%) predominating. Most students were in their second or third academic year (68.2%), and 79.5% reported prior AI use. Notably, only 15.5% had received formal AI education (Table 1).

**Table 1:** Demographic characteristics (*N* = 264)

Variable	*N* (%)	M (SD)
**Age**		21.1 (1.3)
**Gender**		
- Male	160 (60.6%)	
- Female	104 (39.4%)	
**Academic Year**		
- First	39 (14.8%)	
- Second	90 (34.1%)	
- Third	90 (34.1%)	
- Fourth	45 (17.0%)	
**AI Use**		
- Yes	210 (79.5%)	
- No	54 (20.5%)	
**AI Education**		
- Yes	41 (15.5%)	
- No	223 (84.5%)	

### AI anxiety and attitudes

AI anxiety was high (mean = 80.3, SD = 9.4), with the Job Replacement subscale scoring highest (25.7 ± 1.4). Attitudes toward AI were positive overall (mean = 114.3 ± 12.8), particularly in Behavioral Components (53.8 ± 8.2), indicating willingness to engage with AI (Table 2).

**Table 2:** AI anxiety and attitudes scores

Variable	Mean (SD)	Range
**AI Anxiety (Total)**	80.3 (9.4)	21–105
- Learning	24.3 (7.0)	6–30
- Job Replacement	25.7 (1.4)	5–25
- Sociotechnical Blindness	17.5 (1.0)	4–20
- AI Configuration	12.7 (1.4)	6–30
**Attitudes (Total)**	114.3 (12.8)	26–130
- Cognitive	17.5 (3.8)	4–20
- Affective	43.0 (6.7)	10–50
- Behavioral	53.8 (8.2)	12–60

### Correlations and regression

AI anxiety correlated positively with age (*r* = .422, *p* < .001) and negative attitudes (*r* = .658, *p* < .001), but negatively with AI use (*r* = −.190, *p* = .002). Multiple regression (R² = 0.496) identified attitude as the strongest predictor (B = 5.171, *p* < .001), followed by female gender (B = 4.029, *p* < .001) and non-use of AI (B = 4.271, *p* = .005). Academic year and AI education were non-significant (Table 3).

**Table 3:** Predictors of AI anxiety (Multiple regression)

Predictor	B	β	*p*-value	95% CI
Age	-1.312	− 0.186	0.023	[-2.45, -0.18]
Gender (Female)	4.029	0.210	< 0.001	[2.34, 5.72]
Academic Year	-1.110	− 0.112	0.070	[-2.31, 0.09]
AI Use (No)	4.271	0.184	0.005	[1.28, 7.26]
Attitude	5.171	0.670	< 0.001	[3.98, 6.36]

## Discussion

This study extends the Technology Acceptance Model (TAM) by demonstrating that perceived ease of use and usefulness of AI are mediated by sociocultural factors in the Palestinian context [5, 10]. Despite global trends where technological exposure reduces anxiety, our findings reveal that even among AI users, high anxiety persists, a paradox attributable to limited institutional support and gendered access to technology in Palestine [28, 29]. For instance, while 79.5% of participants reported AI use, these interactions were often superficial (e.g., basic apps), lacking structured training to foster confidence. This aligns with regional studies highlighting infrastructural gaps as barriers to meaningful AI engagement [14].

The findings of this study are consistent with recent research by Tarsuslu, Agaoglu, and Bas [16], who found that digital leadership significantly influences AI anxiety and attitude among nurses. Their study demonstrated that organizational support and structured AI training programs were crucial factors in reducing technology-related anxiety, which parallels our finding that attitude is the strongest predictor of AI anxiety. Similarly, their research highlighted the importance of culturally sensitive approaches to AI education, supporting our recommendations for context-specific interventions in Palestinian nursing education.

The high AI anxiety levels (mean = 80.3) contrast with moderate anxiety reported in Turkey and Europe [12, 13], underscoring the unique stressors faced by Palestinian students, such as economic instability and scarce AI resources in curricula [30]. Conversely, the positive attitudes toward AI (mean = 114.3) mirror global findings [24], suggesting Palestinian students recognize AI’s potential despite systemic hurdles. This duality reflects the “optimism-resistance” paradox observed in developing regions, where enthusiasm for technology coexists with apprehension due to uneven access [11].

The particularly high scores on the behavioral component of attitudes (53.8 ± 8.2) indicate that despite experiencing significant anxiety about AI, Palestinian nursing students demonstrate a strong willingness to engage with and learn about AI technologies. This behavioral readiness suggests that students are motivated to overcome their anxieties when provided with appropriate support and training opportunities [30]. This finding is particularly encouraging as it indicates that the high anxiety levels observed in this population may not necessarily translate to avoidance behaviors but rather represent a manageable barrier that can be addressed through targeted educational interventions.

Female students exhibited higher anxiety, consistent with global patterns [31], but in Palestine, this is compounded by cultural norms limiting women’s access to STEM resources [29]. For example, only 34.1% of participants were female, reflecting broader gender disparities in Palestinian nursing education. Younger students’ anxiety may stem from limited early exposure to AI in Palestinian secondary schools, unlike their counterparts in technologically advanced regions [19]. These findings underscore the need for interventions tailored to local gender and age dynamics [17].

### Theoretical implications and framework development

The results of this study contribute to the expansion of the Technology Acceptance Model (TAM) by demonstrating how cultural, socioeconomic, and institutional factors moderate the relationship between perceived usefulness, ease of use, and behavioral intention in resource-constrained contexts [6, 28]. The persistence of high anxiety levels despite positive attitudes suggests that traditional TAM variables may be insufficient for predicting technology acceptance in developing healthcare education environments [15]. Our findings support the need for an enhanced theoretical framework that incorporates contextual moderators such as institutional support, cultural norms, and resource availability [1].

The observed “optimism-resistance” paradox, where students simultaneously express positive attitudes and high anxiety toward AI, suggests that emotional and cognitive responses to technology may operate through separate pathways in culturally distinct contexts [31]. This finding has important implications for understanding technology acceptance in regions where enthusiasm for technological advancement coexists with legitimate concerns about implementation feasibility and cultural appropriateness [8].

### Implications for policy and curriculum design

To address these challenges, we propose: First, AI Literacy Modules: Integrate hands-on AI training (e.g., ChatGPT simulations, diagnostic tools) into nursing curricula to bridge the gap between theoretical awareness and practical competence [18]. Second, Gender-Sensitive Workshops: Partner with organizations like UN Women to create mentorship programs for female students, addressing cultural barriers to technology access [29]. Third, National AI Policy Frameworks: Advocate for the Palestinian Ministry of Health to adopt AI integration guidelines, aligning with its 2030 Digital Transformation Agenda [20]. Fourth, Cross-Institutional Collaborations: Establish partnerships with AI developers (e.g., IBM Watson Health) to provide low-cost training platforms for resource-limited universities [5].

### Strengths, limitations, and future directions

This study’s large sample (*N* = 264) and high response rate (92.6%) enhance validity, while validated instruments ensure reliability. However, the cross-sectional design precludes causal inferences, and convenience sampling limits generalizability beyond Palestine. Additionally, the administration of English-language instruments in a non-English speaking context, despite pilot testing, may have influenced response patterns and requires consideration in future research. Future research should employ longitudinal designs to track anxiety trends post-intervention and expand samples to rural areas with greater technological disparities.

Future longitudinal research should specifically examine the effectiveness of the proposed interventions in reducing AI anxiety while maintaining positive attitudes. Additionally, qualitative studies exploring the cultural and social factors underlying the optimism-resistance paradox would provide valuable insights for developing more effective AI integration strategies in similar contexts.

## Conclusion

This study identifies attitude toward AI as the strongest predictor of anxiety among Palestinian nursing students, with younger age, female gender, and limited AI exposure exacerbating concerns. Contrary to global trends, AI education did not significantly reduce anxiety, likely due to superficial or inconsistent training in current curricula. These findings underscore the urgent need to address sociocultural and infrastructural barriers unique to Palestine, such as gendered access to technology and resource constraints.

To translate findings into action, we propose three targeted interventions: First, Curriculum Integration should prioritize culturally tailored AI literacy modules, co-created with Palestinian healthcare professionals, to reframe AI as a collaborative tool rather than a replacement. Second, Gender-Responsive Training must establish AI mentorship programs for female students, partnering with organizations like UN Women to dismantle access barriers. Third, Policy Advocacy should align nursing education reforms with the Palestinian Ministry of Health’s 2030 Digital Transformation Agenda, securing funding for AI tools and faculty training to ensure sustainable implementation.

Future research should employ longitudinal designs to assess the impact of these interventions and expand sampling to rural areas with greater technological disparities. Additionally, investigation of the specific mechanisms underlying the behavioral readiness observed among students despite high anxiety levels could inform the development of more targeted and effective intervention strategies. By grounding strategies in local realities, Palestinian nursing education can foster AI readiness while mitigating anxiety, ensuring students thrive in evolving healthcare landscapes.

## Electronic supplementary material

Below is the link to the electronic supplementary material.

Supplementary Material 1

## Data Availability

No datasets were generated or analysed during the current study.
