# Supplementary material for: Insights into the relationship between anxiety and attitudes toward artificial intelligence among nursing students
Source: BMC Nurs. 2025 Jul 1;24:812. doi: 10.1186/s12912-025-03490-2 (PMC12211391; doi:10.1186/s12912-025-03490-2)
Supplement: Supplementary file 1 — Supplementary Material 1 [file 12912_2025_3490_MOESM1_ESM.pdf]

Insights into the Relationship between Anxiety and Attitudes toward Artificial Intelligence among Nursing Students

Dear Nursing Students,

Please complete this survey based on your anxiety and attitudes. It will take no more than ten (10) minutes to complete. All your responses will be treated confidentially and will only be used by members of the research team. We will publish the results of the study online for access by the target audience worldwide.

Your participation in this study is voluntary, and you have the right to withdraw from this survey at any time. However, we look forward to your participation, as your contributions will provide us with a better understanding of your anxiety and attitudes toward the application of artificial intelligence applications in nursing education.

For more information about this study or survey, please feel free to contact the research team.

The questionnaire consists of three scales:

1. **Demographic Information:** This scale measures your age, gender, academic year, monthly income, AI use, and AI education.
2. **Artificial Intelligence Anxiety Scale (AIAS):** This scale measures your feelings of anxiety related to learning, job replacement, sociotechnical blindness, and configuration with AI. The scale uses a 5-point Likert scale (e.g., "Strongly Disagree" to "Strongly Agree").
3. **Student Attitudes Toward Artificial Intelligence (SATAI) Scale:** This scale assesses your cognitive, affective, and behavioral attitudes toward AI. The scale uses a 5-point Likert scale (e.g., "Strongly Disagree" to "Strongly Agree").

Thank you for contributing to this important study. Your participation is greatly appreciated.

Best regards,

Research Team

Palestine Ahliya University

I. Demographic Information:

1. Age: ☐ years.

2. Gender:

- Male. ☐
- Female. ☐

3. Academic Year:

- First year. ☐
- Second year. ☐
- Third year. ☐
- Fourth year. ☐

4. Monthly Income:

- Less than 3000 NIS. ☐
- 3000 -4500 NIS. ☐
- More than 4500 NIS. ☐

5. Did You Use Artificial Intelligence Tools?

- Yes. ☐
- No. ☐

6. Did You Education Artificial Intelligence Tools?

- Yes. ☐
- No. ☐

II. Artificial Intelligence Anxiety Scale (AIAS):

Note. The scale uses **1** point for strongly disagree, **2** points for disagree, **3** points for neutral, **4** points for agree, and **5** points for strongly agree.

**Items**	**1**	**2**	**3**	**4**	**5**
**1.** Learning to understand all of the special functions associated with an AI technique/product makes me anxious.					
**2.** Learning to use AI techniques/products makes me anxious.					
**3.** Learning to use specific functions of an AI technique/product makes me anxious.					
**4.** Learning how an AI technique/product works makes me anxious.					
**5.** Learning to interact with an AI technique/product makes me anxious.					
**6.** Taking a class about the development of AI techniques/products makes me anxious.					
**7.** Reading an AI technique/product manual makes me anxious.					
**8.** Being unable to keep up with the advances associated with AI techniques/ products make me anxious.					
**9.** I am afraid that an AI technique/product may make us dependent.					
**10.** I am afraid that an AI technique/product may make us even lazier.					
**11.** I am afraid that an AI technique/product may replace humans.					
**12.** I am afraid that widespread use of humanoid robots will take jobs away from people.					
**13.** I am afraid that if I begin to use AI techniques/products I will become dependent upon them and lose some of my reasoning skills.					
**14.** I am afraid that AI techniques/products will replace someone's job.					
**15.** I am afraid that an AI technique/product may be misused.					
**16.** I am afraid of various problems potentially associated with an AI technique/ product.					
**17.** I am afraid that an AI technique/product may get out of control and malfunction.					

**18.** I find humanoid AI techniques/products (e.g. humanoid robots) scary.					
**19.** I find humanoid AI techniques/products (e.g. humanoid robots) intimidating.					
**20.** I don't know why, but humanoid AI techniques/products (e.g. Humanoid robots) scare me.					
**21.** I am afraid that an AI technique/product may lead to robot autonomy.					

III. Student Attitudes Toward Artificial Intelligence (SATAI):

Note. The scale uses **1** point for strongly disagree, **2** points for disagree, **3** points for neutral, **4** points for agree, and **5** points for strongly agree.

**Items**	**1**	**2**	**3**	**4**	**5**
**1.** I think that it is important to learn about AI in school.					
**2.** AI class is important.					
**3.** I think that lessons about AI should be taught in school.					
**4.** I think every student should learn about AI in school.					
**5.** AI is very important for developing society.					
**6.** I think AI makes people's lives more convenient.					
**7.** AI is related to my life.					
**8.** I will use AI to solve problems in daily life.					
**9.** AI helps me solve problems in real life.					
**10.** I will need AI in my life in the future.					
**11.** AI is necessary for everyone.					
**12.** AI produces more good than bad.					
**13.** AI is worth studying.					
**14.** I think that most jobs in the future will require knowledge related to AI.					
**15.** I want to work in the field of AI.					
**16.** I will choose a job in the field of AI.					
**17.** I would participate in a club related to AI if there was one.					

**18.** I like using objects related to AI.					
**19.** It is fun to learn about AI.					
**20.** I want to continue learning about AI.					
**21.** I'm interested in AI-related TV programs or online videos.					
**22.** I want to make something that makes human life more convenient through AI.					
**23.** I am interested in the development of AI.					
**24.** It is interesting to use AI.					
**25.** I think that there should be more class time devoted to AI in school.					
**26.** I think I can handle AI well.					
